# Solitary Fibrous Tumour of the Parotid Gland: A Case Report and Review of the Literature

**DOI:** 10.1155/2015/741685

**Published:** 2015-07-06

**Authors:** Matthew M. Kwok, Muthukumar Subramaniyan, Sor Way Chan

**Affiliations:** Department of Otolaryngology, Head and Neck Surgery, Western Health, Footscray, VIC 3011, Australia

## Abstract

*Introduction*. Solitary fibrous tumours (SFT) of the parotid gland are a very rare group of spindle-cell tumours with only 28 cases reported in the literature. This review aims to report an additional case of parotid SFT and provide a review of all reported cases of this rare condition. *Case Presentation*. A 26-year-old male presented a 3 cm well-demarcated, slowly enlarging mass which was completely excised, revealing histological and immunohistochemical features of SFT. *Discussion*. Reviews of all reported cases suggest that histology and immunohistochemistry are paramount in the diagnosis of SFT. These features, along with clinical presentation and management of this rare condition, will be discussed.

## 1. Introduction

Solitary fibrous tumours (SFT) are a rare group of spindle-cell tumours primarily affecting the pleura, although various soft tissue locations have been reported in literature [1]. Currently, there are no clearly recognised aetiological factors for their origin and they can include both benign and malignant lesions with metastatic potential [1]. The proportion of SFT in the head and neck region is estimated to be around 6% of all SFT cases [2]. However, SFT occurring in the parotid is very rare, with only 28 cases of SFT of the parotid gland having been reported in the current literature [3], which may have clinical implications on the accuracy of its diagnosis and management.

## 2. Case Report

A 26-year-old male presented with a two-year history of a slowly enlarging right sided swelling in the parotid region. He was otherwise well and asymptomatic.

Clinical examination revealed an approximately 3 × 3 × 1 cm well-demarcated soft parotid lesion.

Ultrasound imaging revealed the presence of a 3.1 × 3.0 × 1.4 cm heterogenous lesion, which was confirmed on magnetic resonance imaging as a 1.5 × 3.0 × 3.0 cm heterogenous lesion in the parotid gland which was hyperintense on T2 imaging. Multiple attempts at fine needle aspirate did not produce any conclusive results.

A right sided superficial parotidectomy was performed and the patient was discharged on the first postoperative day without any complications.

**Figure 1:**
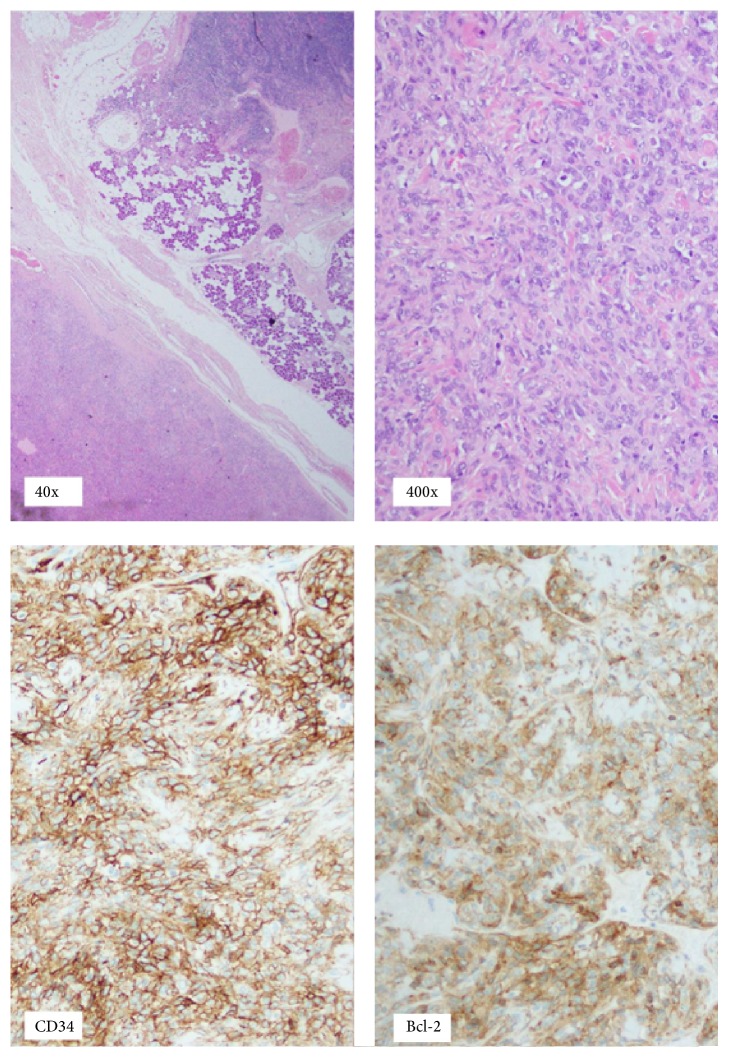
Hematoxylin and eosin staining at low magnification (40x) and high magnification (400x) showing a well circumscribed solid fleshy tan tumour within the parotid gland, with cords and nests of spindle-type cells. Immunoperoxidase staining was positive for CD34 and Bcl-2.

Histopathological examination showed a 2.7 cm well circumscribed solid fleshy tan tumour within the parotid gland, consisting of a uniform appearance of cords and nests of spindle-type cells, surrounded by a thin fibrous capsule. There were scattered intervening eosinophilic dense stroma and some prominent vessels. No evidence of malignancy was observed (Figure 1).

Immunoperoxidase staining was positive for Bcl-2, CD34 (Figure 1), and CD99 and negative for actin, myosin, and S100.

## 3. Discussion

Parotid SFT are a rare type of spindle-cell tumour with only 29 cases of parotid SFT reported in the current literature from 1995 to 2014 (summarised in Table 1). Bauer et al. published the first review of 22 cases of this rare condition in 2010 [1], with Sousa et al. reviewing 3 further cases more recently in 2013 [3].

Clinically, parotid SFT generally present as well-defined, palpable, painless, and slowly growing submucosal masses [1]. There is no predilection for age or gender [1, 4] and this review confirms this with the age of those affected ranging from 11 to 79 years and the male : female ratio being 1 : 2. The duration of symptoms is highly variable and ranges from 2 months to 20 years. Thus, parotid SFT present similarly to other benign parotid tumours and are often difficult to diagnose based on clinical examination.

Likewise, parotid SFT appear nonspecific on radiological imaging, being hypointense with heterogenous contrast enhancement on computed tomography [5], isointense on T1 weighted imaging, and hyperintense on T2 weighted magnetic resonance imaging [1].

Therefore, histology and immunohistochemistry are the most important modalities in the diagnosis of this condition. Macroscopically, parotid SFT appear as well-defined and encapsulated masses with a pale and firm cut surface [1]. The role of fine needle aspiration remains unclear, often with inconclusive results [1].

Microscopic appearances consist of round to spindled cells with tapering cytoplasm arranged in storiform patterns in a collagenous matrix [1, 6]. There may also be variable vascularity with numerous ramifying vessels and hyalinised walls [7]. Malignant invasion of the mandible has been reported in one case of parotid SFT [8].

Immunohistochemistry remains to be the most important modality for confirming the diagnosis of parotid SFT. Markers present in almost all parotid SFT include CD34, bcl-2, vimentin, and CD99. Negative markers used to differentiate against other parotid gland lesions include keratin, EMA, S-100, desmin, smooth muscle actin, muscle-specific actin, smooth muscle myosin heavy chain, and GFAP [1] (Table 2).

The current management of parotid SFT includes complete local excision with clear margins, with long-term follow-up of at least 3 years [1]. The role of adjuvant radiotherapy remains unclear [9]. Our reported case of parotid SFT is consistent with previously reported ranges with regard to patient demographics, duration of symptoms, size, and immunohistological findings.

Due to the rarity of parotid SFT, it is difficult to produce any specific evidence based guidelines for its diagnosis and management. It is therefore important to be aware of this condition in patients presenting with parotid gland lesions in order to provide the appropriate diagnosis and management.

## Figures and Tables

**Table 1:** Summary of all cases of parotid SFT in the current literature.

Year	
Range	1995–2014
Mean	2005
Median	2005
Age	
Range	11–79
Mean	50
Median	46
Gender	
Male	16
Female	13
M : F ratio	1.2
Anatomical location	
Left	15
Right	11
NA	3
Duration of symptoms (months)	
Range	2–240
Mean	45.8
Median	21
NA	6
Size (cm)	
Range	1–18
Mean	4.8
Median	4
NA	2
Management	
Excision	27
Excision and radiotherapy	2

**Table 2:** Immunohistochemical markers in parotid SFT.

Positive	Negative
CD34	Keratin
Bcl-2	EMA
Vimentin	S-100
CD99	Desmin
	Smooth muscle actin
	Muscle-specific actin
	Smooth muscle myosin heavy chain
	GFAP
